# Influenza-Like Illness in Lesotho From July 2020 to July 2021: Population-Based Participatory Surveillance Results

**DOI:** 10.2196/55208

**Published:** 2024-10-08

**Authors:** Abigail R Greenleaf, Sarah Francis, Jungang Zou, Shannon M Farley, Tšepang Lekhela, Fred Asiimwe, Qixuan Chen

**Affiliations:** 1 ICAP at Columbia, 60 Haven Ave, New York, NY, 10032, United States, 1 212 342 0505; 2 Department of Population and Family Health, Mailman School of Public Health, Columbia University, New York, NY, United States; 3 Department of Epidemiology, Mailman School of Public Health, Columbia, New York, NY, United States; 4 Department of Biostatistics, Mailman School of Public Health, Columbia University, New York, NY, United States; 5 Ministry of Health Lesotho, Maseru, Lesotho; 6 Centers for Disease Control and Prevention, Maseru, Lesotho

**Keywords:** surveillance, participatory surveillance, influenza-like illness, COVID-19, cell phone, sub-Saharan Africa, population-based, Lesotho, SARS-CoV-2, technology, epidemiology, adult, data collection, innovation, mobile phone, cellphone

## Abstract

**Background:**

Participatory surveillance involves at-risk populations reporting their symptoms using technology. In Lesotho, a landlocked country of 2 million people in Southern Africa, laboratory and case-based COVID-19 surveillance systems were complemented by a participatory surveillance system called “LeCellPHIA” (Lesotho Cell Phone Population-Based HIV Impact Assessment Survey).

**Objective:**

This report describes the person, place, and time characteristics of influenza-like illness (ILI) in Lesotho from July 15, 2020, to July 15, 2021, and reports the risk ratio of ILI by key demographic variables.

**Methods:**

LeCellPHIA employed interviewers to call participants weekly to inquire about ILI. The average weekly incidence rate for the year-long period was created using a Quasi-Poisson model, which accounted for overdispersion. To identify factors associated with an increased risk of ILI, we conducted a weekly data analysis by fitting a multilevel Poisson regression model, which accounted for 3 levels of clustering.

**Results:**

The overall response rate for the year of data collection was 75%, which resulted in 122,985 weekly reports from 1776 participants. ILI trends from LeCellPHIA mirrored COVID-19 testing data trends, with an epidemic peak in mid to late January 2021. Overall, any ILI symptoms (eg, fever, dry cough, and shortness of breath) were reported at an average weekly rate of 879 per 100,000 (95% CI 782‐988) persons at risk. Compared to persons in the youngest age group (15‐19 years), all older age groups had an elevated risk of ILI, with the highest risk of ILI in the oldest age group (≥60 years; risk ratio 2.6, 95% CI 1.7‐3.8). Weekly data were shared in near real time with the National COVID-19 Secretariat and other stakeholders to monitor ILI trends, identify and respond to increases in reports of ILI, and inform policies and practices designed to reduce COVID-19 transmission in Lesotho.

**Conclusions:**

LeCellPHIA, an innovative and cost-effective system, could be replicated in countries where cell phone ownership is high but internet use is not yet high enough for a web- or app-based surveilance system.

## Introduction

The COVID-19 pandemic required countries to rapidly strengthen their influenza-like illness (ILI) surveillance capacity [1]. Ideally, public health response is guided by real-time surveillance of a disease, but many surveillance systems were not able to quickly pivot to handle an emerging infectious disease such as COVID-19. Given the difficulty of rapidly scaling surveillance systems to monitor COVID-19, the opportunity of widespread access to technologies, such as the internet and mobile phones, as well as the need for surveillance outside of health systems due to stay-at-home orders at the beginning of the pandemic, scientists and governments used creative approaches to surveilling for ILI beyond routine and hospital-based surveillance. Media, electronic, and digital surveillance can be divided into four categories: participatory surveillance, electronic reporting systems, digital surveillance, and event-based surveillance [2].

Participatory surveillance is when a population at risk reports on their health using mobile connectivity, independently of the health care system [3]. Participatory surveillance began as a OneHealth tool in sub-Saharan Africa [4], but in recent years has expanded greatly with COVID-19 [5], among other applications.

In Lesotho, a landlocked country of 2 million people in Southern Africa, laboratory and case-based COVID-19 surveillance systems were complemented by a participatory surveillance system called “LeCellPHIA” (Lesotho Cell Phone Population-Based HIV Impact Assessment Survey) that called participants weekly to report the participants’ and household members’ ILI symptoms. ILI has been shown to be, in multiple settings, an effective proxy measure of COVID-19 [6-8]. This report describes the person, place, and time characteristics of ILI in Lesotho, as created by a participatory surveillance system, from July 15, 2020, to July 15, 2021, and identifies key sociodemographic variables associated with reports of ILI.

## Methods

### Data Source

LeCellPHIA was built from the 2020 Lesotho Population-Based HIV Impact Assessment (LePHIA2020) survey [9]. LePHIA2020 was a cross-sectional, nationally representative household survey that assessed the prevalence of key HIV-related health indicators. The 2-stage cluster survey occurred between December 2019 and March 2020 among adults aged 15 years and older who slept in the house the night before. The sample included 9665 household and 16,468 individual interviews, with a 93.2% household and 93.6% individual interview response rate.

To create the cell phone–based participatory surveillance system, all 342 primary sampling units from the 10 LePHIA2020 districts were included. To ensure we had a sufficient number of older adults in our sample, who were at higher risk of COVID-19 mortality, households with a member aged ≥60 years were oversampled at a ratio of 2:1 between households with and without adults ≥60 years of age. From each sampled household, we randomly selected 1 LePHIA2020 adult participant from among those who consented to future research and provided a valid phone number. Participants who answered the phone call were eligible if they confirmed they participated in LePHIA2020 and lived in the same home where they completed the LePHIA2020 survey or would return in the next year (in which case they were called monthly to check location until they returned and began weekly surveillance calls), confirmed they were ≥18 years of age, provided abbreviated verbal consent, and could participate in English or Sesotho.

### Procedures

Participants were asked “In the past week, have you had any flu like symptoms (eg, fever, dry cough, shortness of breath)?” and if they responded affirmatively, they were asked to specify which symptom they had. Participants were asked the same questions about flu-like symptoms for each household member they had seen in the past week as a proxy report of symptoms. An ILI event was defined as any report of any ILI symptom (ie, multiple symptoms were not required to be counted as ILI). Each participant received a monthly phone credit incentive, varying between US $1 and US $2 based on the number of completed monthly interviews. Further details about implementation are available elsewhere [10].

Beginning in August 2020, weekly weighted estimates of ILI incidence were calculated and shared with stakeholders within a week of data collection. LePHIA2020 household weights were used as the LeCellPHIA base weights, which were adjusted for unequal probability of selection, nonresponse, and potential undercoverage of the sampling frame [11]. The real-time weekly incidence rate was created using the weighted sum of ILI events divided by the weighted sum of all respondents in that week. As the time window was 1 week for all respondents, this percentage was the same as the incidence rate calculation in that week.

### Measures

The procedure for creating the annual weights was the same process as for weekly weight creation, but the annual weights included anyone who participated at least once throughout the year. To calculate the average weekly incidence rate for the year-long period, we included a count of nonconsecutive weeks (ie, the number of unique times a respondent reported symptoms, with at least 2 weeks since the last ILI report) of ILI events in the numerator, and each week a participant reported ILI data in the denominator for themselves and their household members. Since it is possible to have ILI symptoms more than once in a year, a participant was considered at risk again after at least 2 weeks had passed since reporting ILI. The average weekly incidence rate for the year-long period was created using a Quasi-Poisson model, which accounted for overdispersion.

To identify factors associated with increased risk of ILI, we conducted a weekly data analysis by fitting a multilevel Poisson regression model, accounting for three levels of clustering, including enumeration areas, households, and repeated measures, from each person. The predictors included district (Butha Buthe, Leribe, Berea, Maseru, Mafeteng, Mohale’s Hoek, Quthing, Qacha’s Nek, Mokhotlong, and Thaba Tseka), gender (male vs female), age group (15‐19, 20‐29, 30‐39, 40‐49, 50‐59, and ≥60 years), and location (urban, peri-urban, and rural). The model also adjusted for the effect of survey weights by including it as a covariate and the effect of week using a smooth spline with 9 knots.

RStudio (version 2022.7.0.548; Posit) and STATA (version 15.0; Stata Corporation) were used to conduct analyses.

### Ethical Considerations

The Lesotho National Research Ethics Committee and the Columbia University Institutional Review Board approved LeCellPHIA, with exemption from the committee review (AAAT5192). The Centers for Disease Control and Prevention Institutional Review Board reviewed the protocol and deemed the research nonhuman subjects (ID82-2020).

## Results

Interviewers enrolled participants using computer-assisted telephone interview software from July 1 to July 14, 2020, which resulted in a 68% enrollment response rate (American Association for Public Opinion Research Response Rate #2) [12]. Beginning July 15, interviewers asked the same 1776 participants weekly about the participants’ and household members’ ILI symptoms. The response rate for this 12-month period of weekly calls was 78%. Weekly response rates ranged from 68% to 88%. The surveillance system collected data for all weeks in the year-long time period except for the week of December 23‐29, 2020. The LeCellPHIA ILI participatory surveillance system captured 122,985 weekly reports of symptomology in Lesotho between July 15, 2020, and July 15, 2021. We included all observations for participants and household members ≥15 years of age who responded to the ILI symptom question. Primary participants accounted for 51.5% (n=63,381) of the data points, and a proxy report of household member health accounted for 48.5% (n=59,604) of the data. The median age of respondents and household members was 35 (IQR 24‐51) years. Of the 122,985 completed interviews, 693 unique participants had 1085 symptom periods (Table 1). Overall, report of any ILI symptoms (eg, fever, dry cough, and shortness of breath) was at an average weekly rate of 879 per 100,000 (95% CI 782‐988) persons at risk. The weighted average weekly incidence rate of ILI was similar among male (893, 95% CI 761‐1048) and female (867, 95% CI 765‐982) individuals. Among female respondents, the average rate of ILI reports was highest (1053, 95% CI 839‐1322) among persons aged 30‐39 years, whereas among male respondents, the average rate of ILI reports was highest (1124, 95% CI 836‐1510) among persons aged ≥60 years.

**Table 1.** Reports of influenza-like illness symptoms and average incidence rates, by gender, age group, district, and location in Lesotho from July 15, 2020, to July 20, 2021.

Characteristics	Symptomatic reports, n	Total weeks reported, n	Weighted average weekly incidence rate per 100,000 population (95% CI)
**Men**			
**Age group (years)**			
15‐19	33	6708	591 (333‐1051)
20‐29	119	13,852	848 (661‐1058)
30‐39	99	11,386	882 (645‐1089)
40‐49	77	8239	1119 (742‐1205)
50‐59	42	4962	886 (520‐1688)
≥60	125	8926	1124 (836‐1510)
**Total**	495	54,073	893 (761‐1048)
**Women**			
** Age group (years)**			
15‐19	32	7412	410 (267‐629)
20‐29	127	14,192	928 (755‐1140)
30‐39	143	13,761	1053 (839‐1322)
40‐49	88	10,640	808 (585‐1116)
50‐59	67	7789	850 (586‐1233)
≥60	133	15,118	894 (633‐1264)
**Total**	590	68,912	867 (765‐982)
**District**			
Butha Buthe	67	9923	674 (410‐1108)
Leribe	185	20,115	930 (713‐1213)
Berea	109	13,449	777 (542‐1112)
Maseru	353	36,799	949 (788‐1143)
Mafeteng	61	11,870	505 (356‐716)
Mohale’s Hoek	83	8296	1072 (543‐2119)
Quthing	28	5492	430 (228‐812)
Qacha’s Nek	77	5083	1463 (1077‐1989)
Mokhotlong	48	5540	928 (537‐1606)
Thaba Tseka	74	6518	1080 (628‐1857)
**Location**			
Urban	511	54,101	942 (825‐1074)
Peri-urban	110	12,465	952 (609‐1487)
Rural	464	56,419	808 (649‐1007)
**Total**	1085	122,985	879 (782‐988)

**Figure 1.**
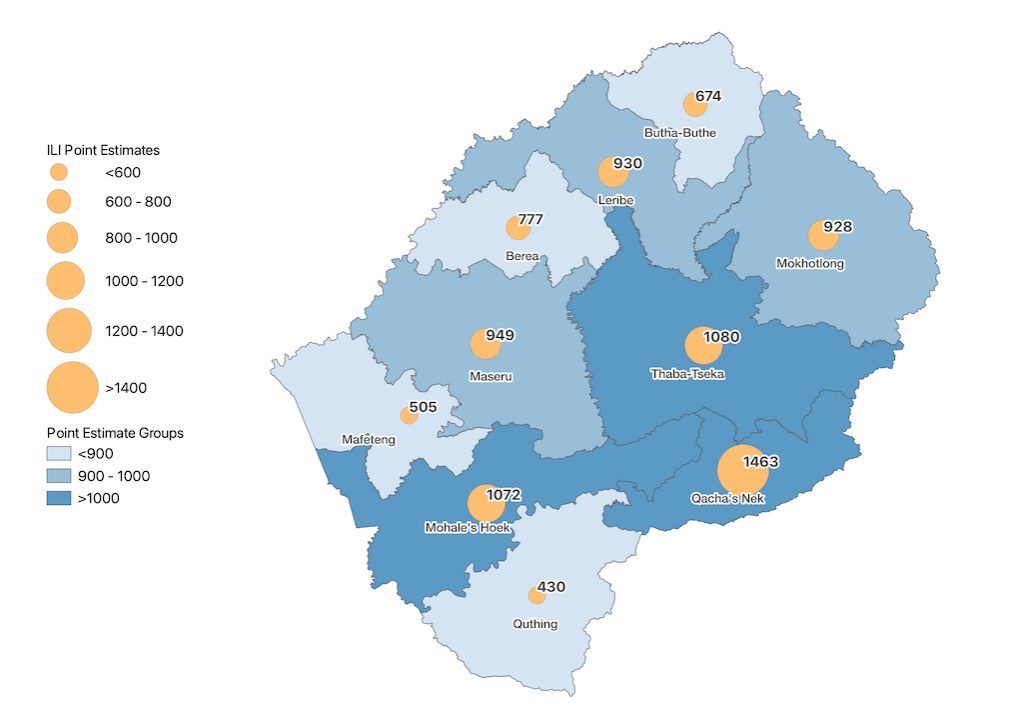
Influenza-like illness (ILI) point estimates by districts in Lesotho from July 15, 2020, to July 20, 2021.

A lower average incidence of ILI was reported in rural (808, 95% CI 649‐1007) compared to urban (942, 95% CI 825‐1074) and peri-urban areas (952, 95% CI 609‐1487). Those in the Qacha’s Nek district had the highest average (1463 per 100,000 population at risk, 95% CI 1077‐1989) incidence rate of ILI, compared to those in the Quthing district, which had the lowest incidence rate (430, 95% CI 228‐812; Figure 1).

**Figure 2.**
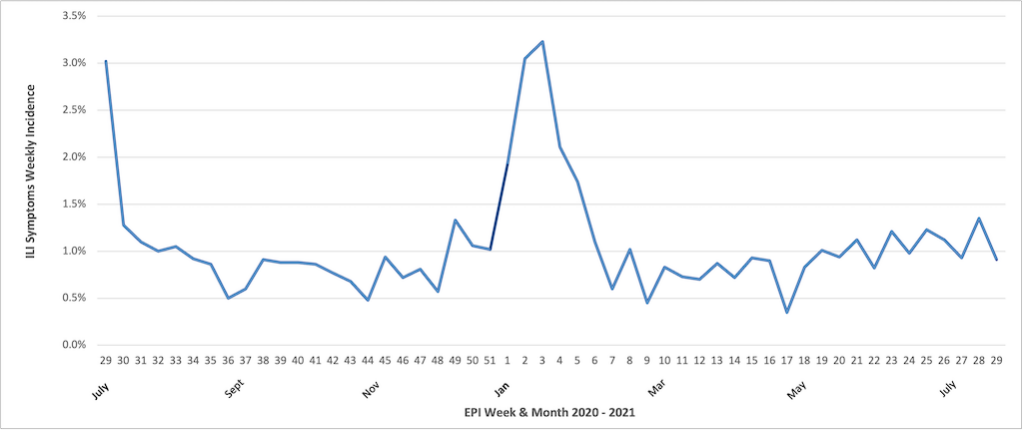
Weekly incidence rate of influenza-like illness (ILI) symptoms reported by the LeCellPHIA (Lesotho Cell Phone Population-Based HIV Impact Assessment Survey) surveillance system. EPI: epidemiological.

The real-time weekly incidence rate of ILI symptoms peaked at 3.3% during the week of January 13‐19, 2021 (Figure 2). The lowest incidence rate (0.35%) occurred in April 21‐27, 2021. Except for the first week of data collection (July 15‐21, 2020, with a 3.02% ILI rate), all weeks with an ILI incidence rate above 1.5% were between December 30, 2020, and February 02, 2021, mirroring the epidemic peak observed in Lesotho’s COVID-19 testing data [13,14].

Table 2 shows the results of the weekly data analysis using multilevel Poisson regression. After accounting for the nonlinear time effect and survey weights, we found that persons residing in the Qacha’s Nek district (risk ratio 2.5, 95% CI 1.5-4.5) and the Thaba Tseka district (risk ratio 1.8, 95% CI 1.1-3.1) had higher risk of ILI compared to persons in the Butha Buthe district. Compared to persons in the youngest age group (15‐19 years), all the individuals in the older age group had elevated risk of ILI, with the highest risk of ILI in the oldest age group (aged ≥60 years; risk ratio 2.6, 95% CI 1.7-3.8).

**Table 2.** Results of the multilevel Poisson regression analysis, adjusting for the nonlinear time effect using a smooth spline on weeks and the survey weights effect.

Characteristics	Risk ratio (95% CI)	
**District**		
Butha Buthe	Reference	
Leribe	1.35 (0.903‐2.125)	
Berea	1.073 (0.679‐1.729)	
Maseru	1.375 (0.902‐2.063)	
Mafeteng	0.772 (0.469‐1.247)	
Mohale’s Hoek	1.509 (0.935‐2.486)	
Quthing	0.858 (0.47‐1.562)	
Qacha’s Nek	*2.545 (1.508‐4.515)^a^*	
Mokhotlong	1.337 (0.752‐2.315)	
Thaba Tseka	*1.837 (1.064‐3.125)^a^*	
**Gender**		
Female	Reference	
Male	1.115 (0.935‐1.363)	
**Age group (years)**		
15‐19	Reference	
20‐29	*1.787 (1.198‐2.597)^a^*	
30‐39	*1.943 (1.282‐2.908)^a^*	
40‐49	*1.966 (1.303‐3.031)^a^*	
50‐59	*1.904 (1.193‐2.989)^a^*	
≥60	*2.606 (1.752‐3.771)^a^*	
**Location**		
Urban	Reference	
Peri-urban	0.748 (0.522‐1.079)	
Rural	0.837 (0.672‐1.03)	

a Italics indicates statistical signficance (*P*<.05).

**Figure 3.**
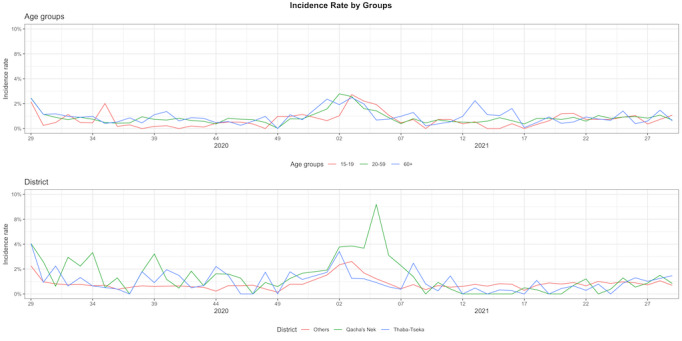
Weekly incidence rate by age group and district in Lesotho from July 15, 2020, to July 20, 2021 (by epidemiological week).

Consistent to the findings in the multilevel Poisson regression, Figure 3 (age groups graph) shows that persons who were ≥60 years of age had the highest estimated incidence rate of ILI, followed by the combined age group of 20‐59 years, and persons who were 15‐19 years of age had the lowest incidence rate of ILI across all weeks. The oldest age group (≥60 years) peaked in epidemiological (EPI) week 1, 2021; those aged 20‐59 years peaked in EPI week 2, 2021; and those aged 15‐19 years peaked in EPI week 3, 2021. Figure 3 (district graph) also displays the weekly estimated incidence rate of ILI in the Qacha’s Nek district, the Thaba Tseka district, and the other 8 districts combined. It shows that the Qacha’s Nek district had a much higher incidence rate of ILI than the other districts in 2020 and early 2021, peaking in week 5 with an incidence rate of 8990 per 100,000 persons. There were also more changes from week to week in the Thaba Tseka district compared to the combined data from the other 8 districts in 2020.

## Discussion

In this study, we described ILI symptoms from July 2020 to July 2021 in Lesotho. Collected by the participatory surveillance system LeCellPHIA, the data mirrored the trends in the COVID-19 pandemic in Lesotho. According to lab data [13], spikes in COVID-19 cases in Lesotho tended to follow increased migration at the border around the Christmas holiday season. LeCellPHIA also recorded spikes in ILI at this time, providing the National COVID-19 Secretariat and other stakeholders with key timely information for public health action and response. Overall, ILI symptoms (eg, fever, dry cough, and shortness of breath) were reported at an average weekly rate of 879 (95% CI 782‐988) per 100,000 persons at risk. ILI was highest among men aged ≥60 years (1124 per 100,000 persons, 95% CI 836‐1510). The oldest age group (≥60 years) had higher risk (risk ratio 2.6) of ILI compared to the youngest age group (15‐19 years) and also reached the epidemic peak before all younger age groups (in January 2021).

LeCellPHIA is a timely participatory surveillance system, with the data collection period running from Thursday to Tuesday each week. The weekly ILI point estimate with a 95% CI was shared on Fridays via email with key stakeholders. LeCellPHIA was created within 6 weeks of receiving funding and collected data through September 2022 (26 months of data collection). Interviewers worked from a confidential, private space in their homes, which allowed LeCellPHIA to function despite lockdowns throughout data collection.

The LeCellPHIA ILI findings are consistent with the COVID-19 trends in lab data reported from Lesotho to the World Health Organization [13]; further analyses found LeCellPHIA and reference standard COVID-19 data had strong correlation, with a Pearson correlation coefficient of 0.67 [14]. With an annual average weekly response rate of 75%, we found that the public in Lesotho—and likely similar contexts in sub-Saharan Africa—is willing to take part in participatory surveillance. LeCellPHIA was rapidly implemented, serving as an example of a sustainable community-based approach that can reach locations without traditional public health surveillance infrastructure.

There are few examples of participatory surveillance systems in low- and middle-income countries (LMICs) that engage the same citizen population weekly over a year; most systems engage community health workers [15], function as a call-in (hotline) system [16], or use an app. Our example of active surveillance by calling a population at risk is unique. Given that LMICs as a whole are less prepared for pandemics [17], continuing to explore the best approach for participatory research that can be rapidly established could offer great benefits in times of public health emergency. Participatory surveillance is being increasingly used in sub-Saharan Africa, outside of One Health programs, providing an example for implementing these systems more broadly for public health [4].

The findings in this report are subject to limitations. Since the majority of COVID-19 cases present asymptomatically, using ILI as a proxy for COVID-19 likely results in an underestimation of COVID-19. Furthermore, given the limited symptom list used, the specificity of the surveillance system is low, as the symptoms could be caused by a number of chronic or infectious diseases. Despite these limitations, LeCellPHIA is a feasible participatory surveillance system that could be replicated in low and middle-income countries where cell phone ownership is adequately high (approximately >80%) to avoid coverage error (ownership is 79% in Lesotho according to LePHIA2020), but internet use is not yet high enough to primarily rely on an app or web-based system.

This study described the rate of ILI reported by cell phone survey participants in Lesotho. It also demonstrated the potential of real-time participatory surveillance via cell phone calls in LMICs. A similar model could be used in other countries, either leveraging existing surveys, as was done for LeCellPHIA, or via random digit dial sampling or calling a sample created from a source such as a government administrative lists, health care facility, or other sources.

Population-based participatory surveillance systems are rare in LMICs. However, LeCellPHIA is an example of a cell phone–based participatory surveillance system that provided data in near real time for public health actors in Lesotho to monitor trends of ILI. The Lesotho National COVID-19 Secretariat used the LeCellPHIA results to guide its COVID-19 risk-adjusted strategy. LeCellPHIA could be replicated in countries where cell phone ownership is high but internet use is not yet high enough for a web- or app-based surveillance system.

## Abbreviations

EPI: epidemiological
ILI: influenza-like illness
LeCellPHIA: Lesotho Cell Phone Population-Based HIV Impact Assessment Survey
LePHIA: Lesotho Population-Based HIV Impact Assessment Survey
LMIC: low- and middle-income country
